# 3,4-DHPEA-EA from *Olea Europaea* L. is effective against standard and clinical isolates of *Staphylococcus sp*.

**DOI:** 10.1186/1476-0711-13-24

**Published:** 2014-07-01

**Authors:** Carlo Bisignano, Angela Filocamo, Giovanna Ginestra, Salvatore V Giofre’, Michele Navarra, Roberto Romeo, Giuseppina Mandalari

**Affiliations:** 1 Dipartimento di Scienze Biologiche ed Ambientali, University of Messina, Salita Sperone 31, 98100 Messina, Italy; 2 Dipartimento di Scienze del Farmaco e Prodotti per la Salute, University of Messina, Viale Annunziata, 98100 Messina, Italy

**Keywords:** *Olea europaea* L, 3,4-DHPEA-EA, Antimicrobial, Staphylococci

## Abstract

**Background:**

The aim of the present work was to evaluate the antibacterial effect of 3,4-DHPEA-EA (methyl-4-(2-(3,4-dihydroxyphenethoxy)-2-oxoethyl)-3-formyl-2-methyl-3,4-dihydro-2H-pyran-5-carboxylate), a derivate of oleuropein, against a range of Gram-positive bacteria, including ATCC strains, food and clinical isolates.

**Methods:**

The minimum inhibitory concentrations (MICs) of 3,4-DHPEA-EA were determined by the broth microdilution method and the Bioscreen C.

**Results:**

3,4-DHPEA-EA was effective against ATCC and clinical isolates of *Staphylococcus aureus* (MIC values between 125 and 250 μg/ml) and ATCC and clinical isolates of *Staphylococcus epidermidis* (MIC values between 7.81 and 62.5 μg/ml). No significant differences were observed between the two solvents (methanol and DMSO) used to dissolve 3,4-DHPEA-EA.

**Conclusions:**

The results obtained could be used to develop novel therapies for the treatment of skin infections. Further studies need to be performed to elucidate the formation of 3,4-DHPEA-EA by acid hydrolysis of oleuropein in the human stomach.

## Background

The potential beneficial effects of biophenols from olives (*Olea europaea* L.) has been observed in several studies, with antioxidant, anti-inflammatory and antimicrobial activities attributed to olive oil [1-3]. Although the health effects of olive oil were traditionally attributed to oleic acid, more recent knowledge has shown that the phenolic fraction plays a crucial role in the reported benefits [4]. Polyphenols that reach the large bowel can beneficially modulate the gut microbial ecosystem increasing the number of *Bifidobacterium* spp., *Lactobacillus* spp. and *Enterococcus* spp. which are known for their anti-inflammatory, immunoregulatory and cholesterol lowering properties through production of short chain fatty acids [5,6]. The most biologically relevant compounds contributing to the sensory and nutritional aspects of olives and olive oil are oleuropein, hydroxytyrosol, quercetin, ferulic acid, caffeic acid, *p*-hydroxybenzoic acid, protocatechuic acid, 3,4-dyhydroxyphenylacetic acid (3,4-DHPA), homovanillic acid and vanilethanediol, whose metabolic and transcriptional profiling has been recently evaluated during fruit development [7]. Oleuropein, present in large quantities in olive tree leaves and in low amounts in extra-virgin olive oil, is known to be responsible for the bitter taste of the oil. We have previously demonstrated that complexes of olive biophenols with β-cyclodextrin were effective decreasing the oil bitterness and preserving from decomposition during storage [8]. The acid-hydrolysis of oleuropein in the stomach generates the formation of a number of metabolites whose distribution and concentration is dependent on the acidity of the gastric compartment [9]. The dyaldehydes originated by the cleavage of the β-glycosidic bond are unstable in the lipid/water interface and are converted into the metabolite known as transposed secoiridoid or dihydropyranic form (3,4-DHPEA-EA) [10].

We have previously demonstrated that aliphatic aldehydes from *Olea europeaea* L. were active against human intestinal and respiratory tract infection strains, whereas *Mycoplasma* spp. were sensitive to oleuropein [11,12]. Other studies have also reported an antibacterial and antifungal action of both olive leaves and olive glutaraldehyde-like compounds [13,14]. A range of microbial gastrointestinal pathogens, including *Escherichia coli* and *Helicobacter pylori*, and viruses such as para-influenza type 3 virus were sensitive to olive oil phenolic compounds [15-17].

The aim of the present work was to investigate the effectiveness of 3,4-DHPEA-EA against a range of Gram-positive bacteria which included ATCC strains and food and clinical isolates.

## Materials and methods

### 3,4-DHPEA-EA

**Figure 1:**
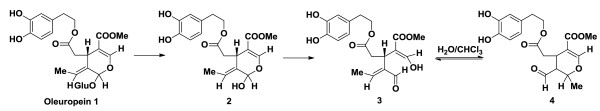
Synthesis of 3,4-DHPEA-EA from oleuropein.

3,4-DHPEA-EA (methyl-4-(2-(3,4-dihydroxyphenethoxy)-2-oxoethyl)-3-formyl-2-methyl-3,4-dihydro-2H-pyran-5-carboxylate) was obtained by enzymatic hydrolysis of oleuropein, as a molecular evolution consequence of the hemiacetal functionality of the aglycon **2**, formed by glycosidic bond cleavage (Figure 1). The lipidic/water interface promotes the rapid rearrangement of the intermediate oleuropeinenol **3** into the final stable biomolecule, the transposed secoiridoid **4**, within 5 min [18].

Thus, to a solution of 100 mg of oleuropein, previously extracted from olive leaves, endogenous β-glucosidase was added at 40°C for 6 h in 20 ml of a H_2_O/CHCl_3_ 1:1 mixture, The mixture was evaporated and purified using Waters XTerra C_18_ column on a Varian HPLC system (H_2_O/MeCN H_2_O/MeCN gradient) with a flow rate of 2 ml/min.

Methyl-4-(2-(3,4-dihydroxyphenethoxy)-2-oxoethyl)-3-formyl-2-methyl-3,4-dihydro-2H-pyran -5-carboxylate was obtained as a yellow oil (20% yield). ^1^H and ^13^C NMR spectra were in agreement with literature data [10].

### Microbial strains and culture conditions

The following Gram-positive strains were used for the antimicrobial testing and were obtained from the University of Messina’s in-house culture collection (Messina, Italy): *Staphylococcus aureus* ATCC 51153, *Staphylococcus aureus* ATCC 6538P, *Staphylococcus aureus* ATCC 43300, *Staphylococcus epidermidis* ATCC 49134, *Staphylococcus epidermidis* ATCC 35984, *Staphylococcus epidermidis* ATCC 12228, *Streptococcus pneumoniae* ATCC 6003, *Streptococcus pyogenes* ATCC 19615, *Streptococcus pyogenes* ATCC 10782, *Listeria monocytogenes* ATCC 7644, *Listeria monocytogenes* ATCC 1392, *Enterococcus hirae* ATCC 10541, *Moraxella catarrhalis* ATCC 8176, 10 food isolates of *L. monocytogenes* belonging to serotypes 1/2a (7 strains) and 1/2b (3 strains), 14 clinical isolates of *S. aureus* obtained from specimens of skin infections and surgical infections, 13 clinical isolates of *S. pneumoniae* obtained from hospitalized patients, 14 clinical isolates of *S. pyogenes* obtained from hospitalized patients, 16 clinical isolates of *M. catarrhalis* obtained from ocular and respiratory tract infections, 13 clinical isolates from *S. epidermidis* obtained from orthopedic protesis, 13 isolates of *E. fecium* and 15 isolates from *E. faecalis* from urinary tract infections.

Cultures for antimicrobial activity tests were grown either in Mueller Hinton Broth (MHB, Oxoid, CM0405, *S. aureus, S. epidermidis, L. monocytogenes, E. hirae, E. fecium, E. faecalis*) or Brain Heart Infusion (BHI, Difco) at 37°C (24 h). For solid media 1.5% (w/v) agar (Difco) was added.

### Antimicrobial testing

The minimum inhibitory concentrations (MICs) of 3,4-DHPEA-EA solubilized in either methanol or dimethyl sulfoxide (DMSO) were determined by the broth microdilution method, according to CLSI [19]. The MICs were also performed in the Bioscreen C (Labsystems Oy, Helsinki, Finland) for all strains as previously reported [20].

All experiments were performed in triplicate on three independent days. A number of positive and negative controls with selected antibiotics (ampicillin and ciprofloxacin) and solvents (methanol, DMSO) were included in each assay.

## Results

### Minimum inhibitory concentrations

The MIC values of 3,4-DHPEA-EA against the ATCC strains tested are shown in Table 1. Results of negative controls indicated the complete absence of inhibition of all the strains tested (data not shown). Analogue values of MICs were obtained with the broth microdilution method and in the Bioscreen C. No differences in the MIC values were recorded with the two solvents utilized (methanol or DMSO). Amongst the Gram-positive bacteria tested, 3,4-DHPEA-EA was active against staphylococci, the most sensitive strains being *S. epidermidis* ATCC 49134 and *S. epidermidis* ATCC 12228, followed by *S. aureus* spp. The effect was bacteriostatic rather than bactericidal.

**Table 1:** Minimum inhibitory concentration (MIC) of 3,4-DHPEA-EA against ATCC Gram-positive bacteria

**Strain**	**3,4-DHPEA-EA**
*S. aureus* ATCC 51153	125
*S. aureus* ATCC 6538P	125
*S. aureus* ATCC 43300	250
*S. epidermidis* ATCC 49134	7.81
*S. epidermidis* ATCC 35984	62.5
*S. epidermidis* ATCC 12228	15.6
*S. pneumoniae* ATCC 6003	> 1000
*S. pyogenes* ATCC 19615	> 1000
*L. monocytogenes* ATCC 7644	> 1000
*L. monocytogenes* ATCC 1392	> 1000
*E. hirae* ATCC 10541	>1000
*M. catarrhalis* ATCC 8176	>1000

Values are expressed as μg ml^-1^ and represent the mean of three determinations.

3,4-DHPEA-EA: (methyl-4-(2-(3,4-dihydroxyphenethoxy)-2-oxoethyl)-3-formyl-2-methyl-3,4-dihydro-2H-pyran-5-carboxylate).

Table 2 reports the MICs of 3,4-DHPEA-EA against the clinical and food isolates tested. MIC values of 7.8 and 15.6 μg ml^-1^ 3,4-DHPEA-EA, respectively, inhibited the growth of 50% and 90% of the *S. epidermidis* strains tested, whereas 125 and 250 μg ml^-1^ 3,4-DHPEA-EA, respectively, inhibited the growth of 50% and 90% of the *S. aureus* strains tested. All the other isolates were resistant. Higher MIC values were obtained with *S. aureus* compared to *S. epidermidis* (Table 2).

**Table 2:** Minimum inhibitory concentration (MIC) of 3,4-DHPEA-EA against food and clinical isolates

**Strain**	**MIC 50**	**MIC 90**
*S. aureus*	125	250
*S. epidermidis*	7.8	15.6
*S. pyogenes*	> 1000	> 1000
*S. pneumoniae*	> 1000	> 1000
*M. catarrhalis*	> 1000	> 1000
*L. monocytogenes*	> 1000	> 1000
*E. faecalis*	> 1000	> 1000
*E. fecium*	> 1000	> 1000

Values are expressed as μg ml^-1^ and represent the mean of three determinations.

3,4-DHPEA-EA: (methyl-4-(2-(3,4-dihydroxyphenethoxy)-2-oxoethyl)-3-formyl-2-methyl-3,4-dihydro-2H-pyran-5-carboxylate).

## Discussion

The present study has evaluated the antimicrobial effect of a metabolite from oleuropein, 3,4-DHPEA-EA, against a range of Gram-positive bacteria, which included ATCC strains, food and clinical isolates. We have recently demonstrated that polyphenols from pistachios had a bactericidal effect against *S. aureus* and *L. monocytogenes*[21], whereas almond skin extracts rich in polyphenols were active against a range of Gram-positive bacteria [22]. The effect on *S. aureus* could be used to find potential applications as a topical treatment for *S. aureus*. Other authors have reported on the antioxidant and antimicrobial activities of individual and combined phenolics in *Olea europea* leaf extract: oleuropein and caffeic acid were active against *Salmonella enteridis*, *Bacillus cereus* and *Escherichia coli* and the antimicrobial effect of the combined phenolics was significantly higher than those of the individual compounds [23]. Using agar dilution and broth microdilution techniques, Sudjana et al. [24] found that a commercial olive leaf extract was active against *Campylobacter jejuni*, *Helicobacter pylori* and *Staphylococcus aureus* with low MIC concentrations (0.31–0.78% v/v). Another investigation on the antimicrobial effect of an olive leaf extract showed that *Bacillus subtilis* was less susceptible than *E. coli*, *Pseudomonas aeruginosa*, *Klebsiella pneumoniae* and *S. aureus*[25]. A commercial olive powder and 4-hydroxytyrosol were able to inactivate *S. aureus* and its enterotoxin A, secreted by the bacteria in 78% of the outbreaks [26]. Although no reports have identified the possible mechanisms of action of the phenolic compounds present in olive leaf, some authors report the activity of phenolics on Gram-positive bacteria may be due to the cell wall or cell membrane disruption together with cell enlargement, which is more susceptible compared to Gram-negative strains [27]. Fabiani et al. [28] demonstrated that hydrogen peroxidase production is responsible for the induction of apoptosis by hydroxytyrosol on HL60 cells.

In our previous study we demonstrated that oleuropein and hydroxytyrosol were active against ATCC strains and clinical isolates: the MIC values of hydroxytyrosol ranged between 0.24 and 7.85 μg ml^-1^ for ATCC strains and between 0.97 and 31.25 μg ml^-1^ for clinically isolated strains, whereas the MIC values of oleuropein ranged between 62 and 500 μg ml^-1^ for ATCC strains and between 31.25 and 250 μg ml^-1^ for clinically isolated strains [3]. Although oleuropein was found effective against a range of Gram-positive strains, in the present work we demonstrated that 3,4-DHPEA-EA, a metabolite obtained by hydrolysis of oleuropein, was only active against ATCC and clinical isolates of *S. aureus* and *S. epidermidis*. The use of *Olea* metabolites could therefore be tested in combination with traditional antibiotics in order to identify new mechanisms of synergism and antibiotic-resistant modulating properties for the development of novel drugs. On the basis of previous investigations on the absorption and metabolism of olive oil secoiridoids in the small intestine [29], further studies need to be performed to elucidate the formation of 3,4-DHPEA-EA in the stomach.

## Conclusions

In summary, the results of the present study showed that a metabolite from oleuropein was effective against staphylococci and could therefore be a potential source of natural antimicrobials for the treatment of skin infections. However, further studies are needed to understand the mechanisms responsible for these activities and the obtained *in vitro* results need to be translated both in food and *in vivo*.

## Competing interest

The authors declare no conflict of interest.

## Authors’ contributions

GM designed research, CB, AF, GG, SVG carried out research, GM, MN, RR analysed results, GM wrote the manuscript. All authors read and approved the final manuscript.
